# Copeptin, Insulin Resistance, and Risk of Incident Diabetes in Older Men

**DOI:** 10.1210/JC.2015-2362

**Published:** 2015-07-09

**Authors:** S. Goya Wannamethee, Paul Welsh, Olia Papacosta, Lucy Lennon, Peter H. Whincup, Naveed Sattar

**Affiliations:** Department of Primary Care and Population Health (S.G.W., O.P., L.L.), University College Medical School, Royal Free Campus, London NW3 2PF, United Kingdom; British Heart Foundation Glasgow Cardiovascular Research Centre (P.W., N.S.), Faculty of Medicine, University of Glasgow, G12 8TA Glasgow, United Kingdom; and Department of Community Health Sciences (P.H.W.), St George's, University of London SW17 ORE, London, United Kingdom

## Abstract

**Context:**

Prior studies suggested a role for the arginine vasopressin (AVP) system in the pathogenesis of diabetes. Prospective studies on the association between copeptin (the C-terminal fragment of AVP hormone) and incident diabetes are limited.

**Objective:**

We have examined the association between plasma copeptin and the risk of incident diabetes in older men.

**Design:**

The British Regional Heart Study was a prospective study with an average of 13 years follow-up.

**Setting:**

General practices in the United Kingdom were studied.

**Participants:**

Participants were 3226 men aged 60 to 79 years with no prevalent diabetes.

**Outcome:**

We measured 253 patients with incident diabetes.

**Results:**

Copeptin was positively and significantly associated with renal dysfunction, insulin resistance (homeostasis model assessment of insulin resistance), metabolic risk factors (waist circumference, blood pressure, triglycerides, and liver function), C-reactive protein, tissue plasminogen activator, and von Willebrand factor (endothelial dysfunction) but not with plasma glucose. The risk of incident diabetes was significantly elevated only in men in the top fifth of the copeptin distribution (>6.79 pmol/L), and this risk persisted after adjustment for several diabetes risk factors including metabolic risk factors and C-reactive protein (adjusted hazard ratio in the top fifth vs the rest = 1.78 [95% confidence interval, 1.34–2.37]). Risk was markedly attenuated although it remained significant after further adjustment for homeostasis model assessment of insulin resistance and plasma glucose (adjusted hazard ratio = 1.47 [1.11–1.97]). The increased risk was seen even when the analysis was restricted to men with no chronic kidney disease or to men with no impaired fasting glucose (<6.1 mmol/L).

**Conclusion:**

Copeptin is associated with a significantly increased risk of diabetes in older men. The association is partly mediated through lower insulin sensitivity. The findings suggest a potential role of the AVP system in diabetes.

Arginine vasopressin (AVP), also termed antidiuretic hormone, is a nonapeptide produced in the hypothalamus. AVP is released from the neurohypophysis to promote renal water conservation, which affects osmoregulation and cardiovascular homeostasis (1, 2). The AVP system has been postulated to play a role in glucose homeostasis, insulin resistance, and lipid and fat metabolism in human and animal studies (3–6). Vasopressin is elevated in patients with type 2 diabetes (7). However, the plasma AVP level is difficult to measure and unreliable because of its instability and short half- life (8). Plasma C-terminal provasopressin (copeptin), the C-terminal part of the AVP precursor peptide, is stable in serum or plasma, making it a more convenient biomarker. Copeptin has been established as a reliable marker of circulating AVP concentration in routine clinical practice (8). Several cross-sectional population studies have shown copeptin to be strongly and positively associated with insulin resistance, obesity and metabolic abnormalities (9–14), and major risk factors for the development of diabetes. Copeptin has shown to be raised in subjects with diabetes although one study suggested that the association is seen in men only (13). Prospective studies on the association between copeptin and the risk of incident diabetes, however, are limited and the results are inconsistent (14–16). Among the 3 prospective studies that have examined the association between copeptin and the risk of incident diabetes, The Malmö Diet and Cancer (MDC) Study showed an association independent of fasting insulin and blood glucose (14), whereas the FINRISK97 Study showed no independent association between copeptin and diabetes after adjustment for metabolic risk factors (16). The Prevention of Renal and Vascular Endstage Disease (PREVEND) Study suggested sex differences in the association between copeptin and diabetes, reporting an independent association in women but not in men (15). Insulin resistance is associated with systemic inflammation and markers of endothelial dysfunction, including tissue plasminogen antigen (tPA) and von Willebrand factor (vWF) (17, 18), which have been related to diabetes incidence (18, 19). The contributing role of these factors to the copeptin-diabetes relationship has not been previously assessed. The aim of this study was to examine the prospective association between copeptin and the risk of diabetes in older men and assess the role of insulin and inflammation and endothelial dysfunction in the association.

## Subjects and Methods

The British Regional Heart Study (BRHS) is a prospective study of cardiovascular disease (CVD) involving 7735 men aged 40 to 59 years, selected from the age-sex registers of 1 general practice in each of 24 British towns, who were screened between 1978 and 1980 (20). In 1998 to 2000, all surviving men, now aged 60 to 79 years, were invited for a 20th year follow-up examination. All relevant local research ethics committees provided ethical approval. All men provided informed written consent to the investigation, which was carried out in accordance with the Declaration of Helsinki. They completed a questionnaire (Q20) that included questions on their medical history and lifestyle behavior. The men were requested to fast for a minimum of 6 hours, during which time they were instructed to drink only water and then to be present for measurement at a specified time between 8:00 amand 6:00 pm. Twelve-lead electrocardiograms (ECGs) were recorded using a Siemens Sicard 460 instrument and were analyzed using the Minnesota Coding definitions at the University of Glasgow ECG core laboratory. All men were asked to provide a blood sample, collected using the Sarstedt Monovette system. A total of 4252 men (77% of survivors) presented for examination.

### Study subjects

Blood measurements of copeptin were available for 3666 men (86%) at the 20-year follow-up examination (1998–2000). Men with a doctor diagnosis of diabetes, and men with a diagnosis of diabetes in the year of reexamination and those with a fasting glucose of >7 mmol/L (World Health Organization criteria) were considered to have prevalent diabetes and were excluded (n = 426). Men taking vasopressin drugs (n = 14) were also excluded. The analysis is thus based on 3226 men.

### Cardiovascular risk factors

Details of measurement and classification methods for smoking status, physical activity, waist circumference (WC), social class, blood pressure (BP), high-density lipoprotein cholesterol (HDL-C), and triglycerides in this cohort have been described previously (21–24). Preexisting CVD in men was defined as a history of a doctor diagnosis of myocardial infarction, stroke, or heart failure. The longest-held occupation of subjects was used to define social class using the Registrar Generals' Social Class Classification: I (professionals), II (managerial), III nonmanual (clerical), III manual (skilled), IV manual (partly skilled), and V manual (unskilled). Manual workers included men in social classes III manual, IV, and V. Heart rate was measured with an ECG. Plasma glucose was measured by a glucose oxidase method using a Falcor-600 automated analyzer (25). Serum insulin was measured using an ELISA that does not cross-react with proinsulin (26). Triglyceride, glucose, and insulin concentrations were adjusted for the effects of fasting duration and time of day (24). Insulin resistance was estimated according to the homeostasis model assessment (HOMA), the product of fasting glucose (millimoles per liter) and insulin (units per milliliter) divided by the constant 22.5 (27). We also calculated the insulin secretory function as quantified by the homeostasis model assessment of β-cell function (HOMA-β) based on fasting insulin and glucose defined as HOMA-β = 20 × insulin (units per milliliter)/(glucose [millimoles per liter) − 3.5) (27). Homeostasis model assessment of insulin resistance (HOMA-IR) and HOMA-β are highly correlated (*r* = 0.81). Predicted glomerular filtration rate (eGFR) (measure of renal function) was estimated from serum creatinine using the equation eGFR = 186 × (creatinine) − 1.154 × (age) − 0.203 (28). Chronic kidney disease was defined as eGFR of <60 mL/min/1.73 m^2^. Plasma levels of tPA antigen were measured with an ELISA (Biopool AB) as was vWF antigen (DAKO) (17). C-reactive protein (CRP) was assayed by ultrasensitive nephelometry (Dade Behring). γ-Glutamyl transferase (GGT) was measured using a Hitachi 747 automated analyzer.

### Laboratory methods

Manufacturers' calibrators and controls were used in the measurement of copeptin in accordance with their instructions. Copeptin was measured using an ultrasensitive method on a BRAHMS Kryptor compact plus (B.R.A.H.M.S.). The lower limit of sensitivity was 0.9 pmol/L. The low control coefficient of variation was 4.7%, and the high control coefficient of variation was 4.6% (23).

### Follow-up

All men have been followed up for all-cause mortality, cardiovascular morbidity, and development of type 2 diabetes from the initial examination to July 2012 (29), and follow-up has been achieved for 99% of the cohort. This analysis is based on follow-up from rescreening in 1998 to 2000, a mean follow-up period of 13 years (12–14 years). Information on deaths was collected through the established “tagging” procedures provided by the National Health Service registers. Evidence regarding diabetes was obtained by reports from general practitioners and by biennial reviews of the patients' notes (including hospital and clinic correspondence) through to the end of the study period.

### Statistical methods

The distribution of copeptin was skewed, and log transformation was used. The men were divided into equal fifths based on the copeptin distribution in all men. The Cox proportional hazards model was used to initially assess the age-adjusted hazard ratio (HR) (relative risk) in a comparison of quintiles of copeptin. Because of a threshold effect of copeptin on incident diabetes, subsequent analyses were performed to adjust for potential confounders and mediators in a stepwise manner, comparing the top fifth vs the rest. In multivariate analyses, smoking (never, long-term ex-smokers [>15 years], recent ex-smokers [<15 years], and current smokers), social class (manual vs nonmanual), physical activity (4 groups), use of antihypertensive treatment (yes/no), statin use (yes/no), and preexisting CVD (yes/no) were fitted as categorical variables. WC, systolic BP, triglycerides, HOMA, GGT, CRP, eGFR, plasma glucose, and vWF were fitted as continuous variables. Receiver operating characteristic curves and areas under the curve (C-statistic) were used to assess the incremental value of copeptin to predict diabetes beyond a basic score, which included routine risk factors for diabetes readily obtainable in clinical practice, eg, age, WC, smoking, hypertension, use of statins, antihypertensive drugs, preexisting CVD, renal function, GGT, triglycerides, and fasting blood glucose. We calculated risk function estimates based on the regression coefficients of the Cox models with and without copeptin in the model. Tests for differences between the C-statistics for the risk function models with and without copeptin were performed using an SAS macro (% receiver operating characteristic curve) with SAS software (version 9.3).

## Results

During the mean follow-up period of 13 years, there were 253 incident diabetes cases in the 3226 men. Table 1 shows the baseline characteristics and biological and metabolic risk markers by quintiles of copeptin. Copeptin was significantly associated with WC, HOMA-IR, BP, blood lipids (HDL-C and triglycerides), CRP, vWF, tPA, eGFR, heart rate, and GGT. No association was seen with plasma glucose, glycated hemoglobin (HbA_1c_), or total or low-density lipoprotein cholesterol.

**Table 1.** Baseline Characteristics by Quintiles of Copeptin in Men With No Prevalent Diabetes

	Copeptin Quintiles					*P*_trend_
	<2.18 pmol/L (n = 647)	2.18–3.12 pmol/L (n = 643)	3.13–4.45 pmol/L (n = 648)	4.46–6.78 pmol/L (n = 642)	6.79 pmol/L (n = 646)	
Age, y	67.7 (5.5)	68.4 (5.3)	68.6 (5.7)	68.9 (5.7)	69.4 (5.4)	<.0001
Body mass index, kg/m^2^	25.9 (3.1)	26.3 (3.4)	26.6 (3.6)	27.0 (3.7)	27.5 (3.9)	<.0001
WC, cm	94.1 (9.4)	95.3 (9.4)	96.4 (9.9)	97.6 (9.9)	98.8 (10.5)	<.0001
% inactive	28.8	32.5	30.2	33.4	40.6	<.0001
% smokers	10.9	12.6	13.9	13.6	13.5	.57
% heavy drinkers	3.2	2.5	3.5	4.7	3.3	.40
% parental history	26.2	27.4	26.7	25.9	25.9	.97
% manual workers	47.3	50.3	54.6	55.8	61.8	<.0001
% statins	6.5	5.9	8.6	6.7	6.3	.33
% antihypertensive drugs	26.6	28.2	28.5	31.6	43.1	<.0001
% preexisting CVD	11.4	13.2	16.2	12.4	17.6	<.0001
Biological markers						
Systolic BP, mm Hg	145.5 (24.1)	146.6 (22.0)	149.2 (23.6)	150.5 (24.5)	149.6 (24.9)	<.0001
Cholesterol, mmol/L	6.04 (1.00)	6.08 (1.18)	6.07 (1.04)	6.08 (1.11)	6.02 (1.11)	.77
Triglyceride, mmol/L	1.44 (1.07–1.97)	1.52 (1.09–2.18)	1.56 (1.12–2.07)	1.63 (1.16–2.17)	1.61 (1.18–2.34)	<.0001
HDL-C, mmol/L	1.35 (0.33)	1.34 (0.34)	1.35 (0.33)	1.34 (0.35)	1.28 (0.34)	.004
Glucose, mmol/L	5.53 (5.21–5.90)	5.53 (5.21–5.87)	5.58 (5.25–5.97)	5.53 (5.19–5.87)	5.58 (5.22–5.96)	.26
HbA_1c_, %	4.85 (0.74)	4.81 (0.55)	4.81 (0.55)	4.86 (0.62)	4.89 (0.64)	.09
Log HOMA-IR	0.56 (0.56)	0.60 (0.58)	0.66 (0.57)	0.69 (0.59)	0.84 (0.64)	<.0001
Heart rate, beats/min	62.82 (11.3)	64.59 (11.7)	65.81 (12.33)	65.17 (11.99)	67.04 (13.51)	<.0001
GGT, U/L	25.5 (18–35)	26.6 (18–36)	27.1 (18–36)	28.8 (19–37)	28.5 (20–40)	.002
CRP, mg/L	1.27 (0.60–2.50)	1.52 (0.74–3.08)	1.46 (0.76–3.02)	1.79 (0.89–3.62)	2.20 (1.05–5.00)	<.0001
vWF, IU/dL	127.1 (41.1)	133.2 (42.7)	137.0 (43.3)	139.8 (43.7)	151.4 (48.2)	<.0001
tPa, ng/mL	9.37 (3.71)	10.39 (3.94)	11.00 (4.19)	11.14 (4.18)	11.54 (4.68)	<.0001
eGFR, mL/min/1.73 m^2^	76.6 (11.0)	74.9 (13.0)	74.0 (11.1)	72.4 (13.0)	67.1 (14.1)	<.0001
% eGFR <60	7.3	8.7	9.1	15.1	28.1	<.0001

Data are means (SD) or geometric means (interquartile range).

a *P*_trend_ across groups.

Table 2 shows correlations between copeptin and biomarkers shown to be associated with copeptin in the univariate analysis, adjusted sequentially for age, WC, and HOMA-IR. In age-adjusted analyses, copeptin was significantly associated with WC, HOMA-IR, HOMA-β, systolic BP, HDL-C, triglycerides, eGFR, GGT, CRP, vWF, and tPA. With the exception of HDL-C, copeptin remained significantly associated with these factors after adjustment for age, WC, and HOMA-IR.

**Table 2.** Spearman Partial Correlation Coefficients Between Copeptin and Biological Markers

	Age Adjusted	Age and WC Adjusted	Age + WC + HOMA-IR adjusted
WC	0.15^a^		
HOMA-IR	0.16^a^	0.08^a^	
HOMA-β	0.14^a^	0.08^a^	
Systolic BP	0.08^a^	0.04^b^	0.04^b^
Triglyceride	0.11^a^	0.06^c^	0.04^b^
HDL-C	−0.06^c^	−0.02	0.003
GGT	0.09^a^	0.07^c^	0.06^b^
CRP	0.16^a^	0.13^a^	0.12^a^
vWF	0.18^a^	0.17^a^	0.16^a^
tPa	0.15^a^	0.11^a^	0.09^a^
eGFR	−0.29^a^	−0.26^a^	−0.28^a^

a *P* < .0001.

b *P* < .01.

c *P* < .001.

**Figure 1.**
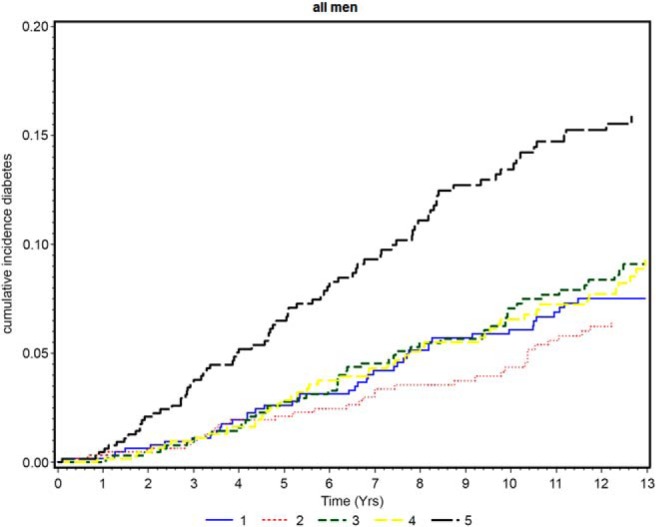
Kaplan-Meier curve of cumulative diabetes incidence by quintiles of copeptin in 3226 men without prevalent diabetes.

Figure 1 shows the Kaplan-Meier estimates of the cumulative incidence of diabetes by fifths of copeptin. The risk of diabetes was higher only in those in the top fifth of the copeptin distribution. The age-adjusted relative risks (95% confidence interval) for the 5 copeptin groups were 1.00 (reference), 0.86 (0.55–1.34), 1.30 (0.87–1.95), 1.12 (0.74–1.70), and 2.23 (1.54–3.22) across increasing fifths. Because risk was only elevated in the top quintile, we have presented the adjusted relative risk for the top fifth vs the rest of the cohort in Table 3. The higher diabetes risk associated with elevated copeptin remained after adjustment for WC, lifestyle characteristics, social class, renal function (eGFR), use of antihypertensive treatment, statin use, systolic BP, GGT, triglycerides, CRP, and vWF. Inclusion of tPA instead of vWF in the model made no difference. Adjustment for HOMA-IR attenuated the diabetes risk further, but it remained significantly increased. HOMA-IR and HOMA-β are highly correlated, and adjustment for HOMA-β instead of HOMA-IR yielded identical results. Further adjustment for fasting glucose also markedly attenuated the risk although the higher risk was still significant. Additional adjustment for heart rate made no difference to the HR. The association remained significant even after further adjustment for HbA_1c_ in model 5 (adjusted HR = 1.43 [1.06–1.93]). The increased risk was seen even after exclusion of men with evidence of chronic kidney disease (eGFR of <60 mL/min/1.73 m^2^; n = 472 (HR adjusted for variables in model 5 = 1.53 [1.10–2.11]) or after exclusion of men with preexisting CVD (myocardial infarction, heart failure, or stroke; n = 454) (adjusted HR = 1.40 [1.01–1.94]). When examined separately in those with and without impaired fasting glucose (IFG) (≥6.1 mmol/L), copeptin was strongly associated with risk of diabetes in both groups after adjustment for age. The association between copeptin and incident diabetes was somewhat stronger in those without IFG at baseline after adjustment for HOMA-IR and metabolic risk factors (1.56 [1.04–2.34] vs 1.31 [0.82–2.07]) (Table 3). However, a formal test for interaction was not statistically significant (*P* = .25).

**Table 3.** Elevated Copeptin and Adjusted HR (95% CI) of Incident Diabetes (Top Quintile vs the Rest)

	Adjusted HR (95% CI)	
	<6.79 pmol/L	≥6.79 pmol/L
All men		
No. of men	2580	646
Rate/1000 person-years (n)	6.0 (174)	12.2 (79)
Model 1	1.00	1.81 (1.36–2.40)
Model 2	1.00	1.78 (1.34–2.37)
Model 3	1.00	1.61 (1.21–2.17)
Model 4	1.00	1.50 (1.11–2.01)
Model 5	1.00	1.47 (1.10–1.97)
No IFG		
No. of men	2153	515
Rate/1000 person-years	4.4 (106)	7.2 (37)
Age-adjusted	1.00	1.73 (1.19–2.51)
Model 1	1.00	1.63 (1.10–2.42)
Model 2	1.00	1.69 (1.13–2.53)
Model 3	1.00	1.56 (1.04–2.34)
With IFG		
No. of men	418	129
Rate/1000 person-years	14.3 (66)	31.1 (41)
Age-adjusted	1.00	1.86 (1.26–2.76)
Model 1	1.00	1.39 (0.88–2.18)
Model 2	1.00	1.31 (0.83–2.08)
Model 3	1.00	1.31 (0.82–2.07)

IFG = ≥6.1 mmol/L). Model 1: adjusted for age, WC, cigarette smoking, physical activity, social class, use of statins, antihypertensive drugs, systolic BP, and eGFR. Model 2: model 1 + GGT + triglyceride + CRP + vWF. Model 3: model 2 + HOMA-IR. Model 4: model 2 + plasma glucose. Model 5: model 2 + HOMA-IR + plasma glucose.

We also examined whether copeptin improved prediction of diabetes beyond routine clinical parameters. The C-statistic for a model that included age, WC, smoking, hypertension, use of statins, antihypertensive drugs, preexisting CVD, renal function, GGT, triglycerides, and fasting blood glucose was 0.779 (95% confidence interval [CI], 0.749–0.809). There was no improvement in the C-statistic when copeptin was added to the model (C-statistic = 0.779 [95% CI, 0.749–0.809]; *P* [improvement] = .58).

## Discussion

In this study of older men, elevated plasma copeptin, a surrogate marker for plasma AVP release, was significantly associated with a higher risk of incident diabetes independent of established diabetes risk factors. Our findings confirm a previous report of an association between copeptin and incident diabetes independent of fasting insulin and fasting blood glucose (14) and extends this to older adults aged >60 years. We were able to examine a wide range of established risk markers and other relevant pathways including hepatic enzymes (marking excess liver fat) and markers of endothelial dysfunction not previously assessed. The association between elevated copeptin levels and risk of diabetes was, to a modest extent, mediated by insulin resistance.

Data examining the prospective association between copeptin and incident diabetes are limited, and the findings have been inconsistent. The MDC Study in Sweden showed a significant positive association between copeptin and incident diabetes even after adjustment for fasting insulin and fasting blood glucose. In contrast, in the PREVEND Study, a significant association was seen between copeptin and risk of diabetes, which was abolished after adjustment for components of the metabolic syndrome including WC, blood glucose, blood lipids, and hypertension in men. A positive relationship persisted in women only. In the FINRISK97 Study, a positive association was seen between copeptin and diabetes after adjustment for established traditional diabetes risk factors including glucose, but the trend was not significant (16). However, glucose metabolism and related pathways may be mechanisms by which copeptin increases diabetes risk and the specific effects of these adjustments were not shown.

Consistent with other studies, we have observed strong cross-sectional associations between copeptin and eGFR, liver enzymes, insulin resistance, and a cluster of cardiometabolic risk factors including hypertension, abdominal obesity, and the dyslipidemia characteristic of insulin resistance (high triglycerides and low HDL-C) (9–13). Copeptin was strongly associated with heart rate (a marker of increased sympathetic nervous system) and was also strongly correlated with CRP (inflammation) and vWF (a marker of endothelial dysfunction), factors shown to be related to insulin resistance (17) and diabetes (18, 19). However, copeptin correlated weakly with glucose levels and HbA_1c_. The relationship between copeptin and diabetes was to a large extent explained by insulin resistance, but there remained a significant independent association even after adjustment for HOMA-IR and fasting plasma glucose. Adjustment for insulin secretory function as quantified by HOMA-β instead of HOMA-IR yielded identical results. As in the MDC Study, we also observed somewhat stronger associations between copeptin and incident diabetes in those without IFG after adjustment for insulin resistance (14). This was also observed in the PREVEND Study in women (16). Thus, mechanisms other than glucose metabolism and insulin resistance may be important for explaining this relationship. A possible explanation for this finding may relate to enhanced plasminogen activator inhibitor-1 (PAI-1) activity, which has been shown to be predictive of diabetes independent of insulin resistance (30). It is well documented that copeptin is associated with renal dysfunction, as was seen in this study (31). There is evidence that mild or moderate renal insufficiency is associated with activation of inflammation and endothelial dysfunction (32). This is consistent with the findings that copeptin correlated significantly with inflammation and with vWF and tPA (markers of endothelial dysfunction) independently of insulin resistance. Up-regulation of proinflammatory cytokines leads to disturbances in the normal function of the vascular endothelium reflected by increased secretion of endothelium-derived products such as vWF and PAI-1, which have been shown to be predictive of diabetes (18, 19, 30). The relation of PAI-1 to incident diabetes has been shown to be stronger in subjects with normal glucose tolerance status independent of insulin resistance (30), broadly consistent with the findings seen for copeptin, and it may be that copeptin and related perturbances may be more relevant to early development of insulin resistance before meaningful hyperglycemia is evident. The lower attenuation of the association of copeptin with diabetes risk by adjustment for HOMA-IR in men without IFG compared with those with IFG (Table 3) is consistent with this possibility. The association between copeptin and risk of incident diabetes in the present study was weaker than that observed in the MDC Study (14) possibly because women were included in their study, and there is the suggestion that the association between copeptin and diabetes is stronger in women (15). The lack of association seen in men in the FINRISK97 Study and the PREVEND Study after adjustment may relate to the age difference as the subjects in these 2 studies were younger (average age of ∼50 years) compared with subjects in the BRHS (average age of 69 years) and the MDC Study (average age of 59 years).

AVP is a key neurohormone that is synthesized in the hypothalamus. AVP exerts its effect through 3 types of receptors namely the V1 (vasoconstriction and myocardial hypertrophy), the VIb (release of adrenocorticotropic hormone), and the V2 receptor (water retention in the renal collecting duct) (33). Several lines of evidence support a role for AVP in the pathogenesis of diabetes. Copeptin has been suggested as a marker of individual stress (34). Activation of the hypothalamic-pituitary-adrenal axis by AVP (copeptin) in chronic stress may be one of the mediators of its association with adiposity and insulin resistance, given the well-established link between glucocorticoids and glucose dysregulation. AVP also activates V1b receptors on the α-cells of the pancreatic islets to increase the secretion of glucagon and potentiate insulin release from the β-cells of the pancreatic islets (5). With aging, the hypothalamus and pituitary are less sensitive to negative feedback from cortisol and both ACTH and cortisol levels rise with age (35). It is possible that older adults are more sensitive to the hypothalamic-pituitary-adrenal axis reactivity to psychological stress than younger adults. This may explain the positive association seen in this study of older men and lack of association in the PREVEND Study of younger men (15) after adjustment for metabolic risk factors. AVP has a central role in the regulation of water balance and AVP secretion per unit change in plasma osmolality increases with aging (36). Copeptin in older adults may also reflect low water intake, which has been linked to the development of hyperglycemia (37). Finally, it is interesting to note that elevated natriuretic peptides (which cause natriuresis and diuresis) appear to protect against diabetes as we reported using a genetic approach (38), whereas this study demonstrates that high levels of copeptin (indicating that AVP may be causing antidiuretic effects and vasoconstriction) increase diabetes risk. These findings further underline the potential novel links between cardiac-related signals/natriuretic pathways and diabetes risk.

### Strengths and limitations

The study population is socially representative of the United Kingdom, and the follow-up rates in the BRHS are exceptionally high. Further, we had excellent phenotyping, enabling us to adjust comprehensively for a range of diabetes risk factors. The association between copeptin and diabetes may be confounded by other factors such as diet and sleep deprivation. Adjustment for dietary intake of fiber based on self-reporting, which has been shown to be related to diabetes in this study (39), made no difference to the findings. However, we did not have direct measures of diet and we did not have measures of cortisol or sleep deprivation, which may influence diabetes risk (40). Future studies relating sleep disturbance and other dietary markers would be useful to examine to what extent the relationship of copeptins with diabetes was influenced by such factors. Our study was carried out in an older predominantly white male population and we cannot generalize our findings to women, younger men or other ethnic groups. Diabetes incidence in this study relied on documented doctor-diagnosed cases of diabetes, which would inevitably result in underascertainment of cases. Our measure of insulin resistance was based on the standard, validated HOMA method (27). However, the absence of direct measures of insulin resistance (eg, based on glucose clamp methods) could have led to an underestimation of the true relationship between copeptin and incident diabetes.

In conclusion, copeptin was strongly associated with renal dysfunction, adiposity, insulin resistance, metabolic risk factors, and markers of inflammation and endothelial dysfunction. High copeptin levels are associated with significantly higher risk of diabetes in older men which is to some extent mediated through its effect on insulin and related metabolic pathways. However, copeptin did not improve the prediction of incident diabetes beyond prediction models with routine clinical parameters. The totality of findings supports a potential role of the AVP system in the pathogenesis of diabetes. Further studies using a Mendelian randomization approach (which uses genetic single nucleotide polymorphisms linked to specific biomarker changes as instruments to help interrogate causality) could be helpful in examining whether there is a direct causal association between copeptin and insulin resistance and copeptin and type 2 diabetes.
